# Numerical Study on Electromechanics in Cartilage Tissue with Respect to Its Electrical Properties

**DOI:** 10.1089/ten.teb.2018.0214

**Published:** 2019-04-16

**Authors:** Abdul Razzaq Farooqi, Rainer Bader, Ursula van Rienen

**Affiliations:** ^1^ Institute of General Electrical Engineering, Faculty of Computer Science and Electrical Engineering, University of Rostock, Rostock, Germany.; ^2^ Research Laboratory for Biomechanics and Implant Technology, Department of Orthopedics, Rostock University Medical Center, University Medicine Rostock, Rostock, Germany.; ^3^ Department Life, Light & Matter, University of Rostock, Rostock, Germany.

**Keywords:** cartilage, electrical properties, streaming potential, tissue engineering, electromechanical transduction, electrical stimulation

## Abstract

Hyaline cartilage undergoes many substantial age-related physiochemical and biomechanical changes that reduce its ability to overcome the effects of mechanical stress and injury. In quest of therapeutic options, magnetic stimulation and electrical stimulation (ES) have been proposed for improving tissue engineering approaches for the repair of articular cartilage. The aim of this study is to summarize *in silico* investigations involving induced electrical properties of cartilage tissue due to various biophysical stimuli along their respective mathematical descriptions. Based on these, a preliminary numerical study involving electromechanical transduction in bovine cartilage tissue has been carried out using an open source finite element computational software. The simulation results have been compared to experimental results from the literature. This study serves as a basis for further *in silico* studies to better understand the behavior of hyaline cartilage tissue due to ES and to find an optimal stimulation protocol for the cartilage regeneration. Moreover, it provides an overview of the basic models along with mathematical description and scope for future research regarding electrical behavior of the cartilage tissue using open source software.

**Impact Statement:**

The presented research summarizes the basic models with mathematical description regarding electrical behavior of the cartilage tissue. A preliminary numerical study involving electromechanical transduction in bovine cartilage tissue sample has been carried out using an open source finite element software. This research will provide scope for future research regarding electrical behavior of the cartilage tissue using open source software.

## Introduction

**FIG. 1.**
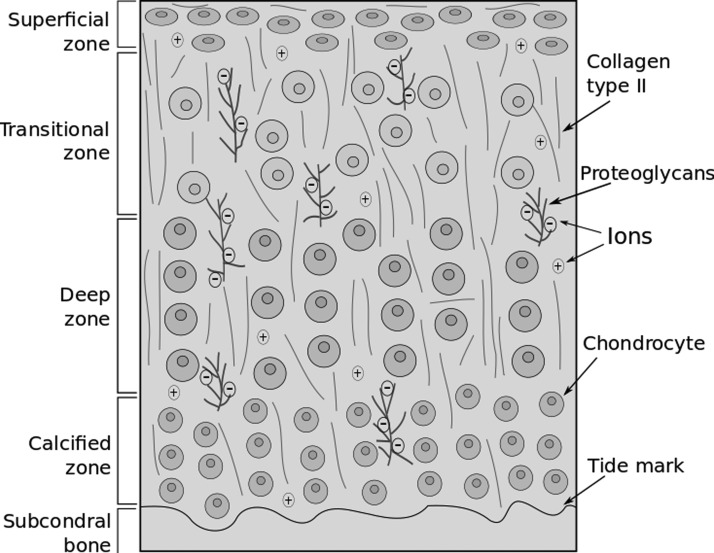
Schematic illustration of composition and structure of articular cartilage lining the bone adapted from Servin-Vences *et al.*^15^ (not drawn to scale).

Articular cartilage is a bradytrophic and avascular tissue that is synthesized and maintained by chondrocytes. Articular cartilage consists of two distinct phases: a fluid phase composed of water and electrolytes, and a solid phase, composed of chondrocytes, collagen fibrils, proteoglycans, and other glycoproteins.^1^ Generally, 60–80% of total wet weight of the articular cartilage is fluid phase and the remaining 20–40% is the solid phase.^2^ Furthermore, collagen fibrils, particularly type II, make up ∼50–75% and chondrocytes less than 5–10% of the solid phase, respectively,^3^ while proteoglycans and other glycoproteins compose most of the remaining solid phase.^4^ Articular cartilage is divided into four zones: superficial, transitional, deep, and calcified zone. Each zone has varying matrix composition, morphology, and cellular and metabolic properties.^5^ The basic structure of the articular cartilage is schematically illustrated in Figure 1.

Mechanics of cartilage and chondrocytes is a function of complicated load sharing between musculoskeletal joints of the body, individual tissue structures within the joint, organization and properties of cells, and the microstructure within the tissue.^6,7^ Damage of the cartilage tissue may lead to osteoarthritis resulting in pain and limitation of mobility for many patients. Treatment of the cartilage defects poses a clinical challenge owing to the lack of intrinsic regenerative capacity of the cartilage. Current therapies available for cartilage repair provide mainly palliative treatment.^8^

Osteoarthritis is characterized by the change in structure and compositional shift of water, solid matrix, and electrolytes in the tissue.^9,10^ Particularly, the fibril-reinforced models of articular cartilage can be used to separate the collagen, proteoglycan, and water content in the tissue to study the cartilage degeneration at the cell and tissue level.^9,11–14^ These studies for the compositional change in the articular cartilage can further be extended using, for example, open source software to include electrical stimulation (ES) for treatment of the cartilage tissue.

Mechanical, magnetic, and electrical stimuli can promote growth, differentiation, and maturation of the cartilage tissue.^16^ Articular cartilage exhibits electromechanical properties due to the electrically charged nature and depth-dependent properties of the tissue.^17^ The mechanical deformation that occurs in cartilage tissue during weight bearing produces internal electrical signals through streaming potentials, by the flow of charged particles across negatively charged proteoglycans of the tissue.^16^ Conversely, applying an external electrical signal to the cartilage tissue can produce stress and deformation in the tissue.^18,19^ Upon removal of the external electrical or mechanical excitation, the deformed cartilage tissue recovers to its initial dimensions, due to the elasticity of the solid matrix, increased osmotic pressure, and fluid redistribution within the tissue.^1,20–22^ Thus, externally applied electrical signals that resemble the endogenous electrical fields have the promising potential to treat osteoarthritis patients in a less invasive manner.

The simplest way of delivering ES is the direct coupling, that is, the cartilage tissue is in contact with the electrodes.^23^ For the indirect ES, two mechanisms have been discussed in literature. The first one is the capacitive coupling^24^ and the second are pulsed electromagnetic fields,^25^ termed as ES and magnetic stimulation (MS), respectively. The MS can be considered a subtype of inductive coupling where the stimulus is delivered in pulses to resemble, for example, the natural strain-generated potentials observed in bone.^26^

Extensive studies have been carried out for the *in silico*, *in vitro*, and *in vivo* investigation of mechanical stimulation on articular cartilage.^5,27–31^ However, few studies are available for MS and ES. Specifically, MS of cartilage has been investigated more in comparison to ES. The limited research available for MS and ES mainly consider the effect of these stimuli for *in vitro* animal and human samples and for *in vivo* animal models. So far, only few simple *in silico* studies have been conducted for these two stimulation types. Yet, *in silico* investigation of these modes of stimuli for cartilage regeneration is required to obtain an optimal strategy for the tissue engineering of neocartilage.^16^

Computational models of human musculoskeletal systems are a valuable tool for studying biomechanics in healthy population, as well as the effects of injury and disease and their respective treatments. Moreover, a computational approach has the power to simulate various surgical conditions without damaging the specimen and to serve for cross validation with experimental models, elucidating important limitations *in vitro*.^32^

In this study, we carried out an analysis of the literature for *in silico* investigations of the induced electrical properties of cartilage. We report on a preliminary numerical study focusing on the linear electromechanical transduction in cartilage tissue using the open source platform FEniCS,^33^ comparing to the results of the experimental study by Frank and Grodzinsky.^34^ In Continuum Mechanics: General Framework for *In Silico* Modeling of Cartilage Tissue Properties section, we outline the general framework of continuum mechanics, which is used for mathematical modeling of the induced electrical properties of cartilage. In Electrical Properties of Cartilage for *In Silico* Studies section, we present a detailed literature survey for *in silico* electrical properties of cartilage induced due to different biophysical stimuli. We describe the novel implementation of the linear electromechanical transduction in a cartilage sample using FEniCS in One-Dimensional Electrokinetic Transduction Model Based on Continuum Theory section.

## Continuum Mechanics: General Framework for *In Silico* Modeling of Cartilage Tissue Properties

Using continuum mechanics, poroelastic and mixture formulations are commonly employed to describe the mechanics and electrical properties of connective tissues like cartilage.^35^ In the poroelastic model, material is considered a porous elastic solid, saturated by the pore fluid that flows relative to the deforming solid. This model is further classified into porohyperelastic,^35^ poroviscoelastic,^36^ and poroviscohyperelastic models.^37^ In the mixture model, material is considered a continuum mixture of a deformable solid phase and a fluid phase. This model is further characterized as biphasic,^20^ triphasic,^38^ and quadphasic,^39^ and then generalized to a multiphasic model.^40^

The biphasic theory models the tissue as a homogeneous mixture of two phases: a charged porous permeable solid phase (collagen-proteoglycan matrix) and an interstitial fluid phase.^41^ In triphasic theory, two phases are same as in the biphasic model, and third, an ion phase with two species (anion and cation) is added.^38^ The quadphasic theory considers the ionic phase further divided into cation and anion phases,^24^ and finally, the multiphasic theory consists of multielectrolytes in addition to a solid and a fluid phase.^40^ When applied to biomechanical studies, the poroelastic and biphasic mixture models are equivalent.

Although most of the continuum mechanics formulations mentioned have been used in literature to discuss the induced electrical properties of cartilage, the triphasic theory is considered the most appropriate for *in silico* description of the induced electrical properties of cartilage tissue due to various biophysical stimuli.^42^

We present the general mathematical formulation for the *in silico* description of induced electrical properties of cartilage applicable to all the mixture theories starting from the biphasic up to the generalized multiphasic theory. Subsequently, some modifications of the triphasic theory used for the investigation of induced electrical properties are presented.

Following the approach developed by Lai *et al.*^38^ and Gu *et al.*,^43^ we model a charged hydrated soft tissue as a continuum mixture consisting of solid phase, interstitial fluid phase, and *k* different species of monovalent or multivalent ions. Thus, there are a total of *k* + 2 constituents in the tissue. Any component other than water and mobile ions within a tissue is considered the solid phase in this model. Unless stated otherwise, *α* = *s*, *w*, *k* always represents solid, fluid, and ionic constituents of the mixture, respectively, in the discussion to follow. For the case of biphasic theory (*α* = *s*, *w*) and for the triphasic and multiphasic theories (*α* = *s*, *w*, *k*), where *k* = 1, 2, 3,…,*n* are *n* ionic species of the mixture. The true mass density $\rho _T^ \alpha$for a component *α* in the mixture is given by

\begin{align*}
\rho _T^ \alpha = { \frac { { m^ \alpha } }  { { V^ \alpha } } } , \tag { 1 } 
\end{align*}

where ${m^ \alpha }$ is the mass of component *α* and ${V^ \alpha }$ is the true volume of phase *α*. The local apparent mass density ${ \rho ^ \alpha }$is defined as follows:

\begin{align*}
{ \rho ^ \alpha } = \frac { { { m^ \alpha } } }  { V } , \tag { 2 } 
\end{align*}

where *V* is the total volume of the mixture. The volume fraction ${ \phi ^ \alpha }$ of each phase *α* is given by

\begin{align*}
{ \phi ^ \alpha } = \frac { { { V^ \alpha } } }  { V } . \tag { 3 } 
\end{align*}

If *α* is w, then ${ \phi ^w}$ is the porosity, which is the volume fraction of the extrafibrillar water available to fixed charges on the proteoglycans.^44^ If *α* is s, then ${ \phi ^s}$ is solidity of the tissue. From Equations (1) to (3), ${ \rho ^ \alpha }$ can be related to the true density $\rho _T^ \alpha$ by

\begin{align*}
{ \rho ^ \alpha } = { \phi ^ \alpha } \rho _T^ \alpha . \tag{4}
\end{align*}

The apparent densities of ion species can also be written as follows:

\begin{align*}
{ \rho ^k} = {c^k}{M^k}{ \phi ^w} \tag{5}
\end{align*}

where *c^k^* and *M^k^* are the concentration and molar weight of the *k*th ionic species, respectively, and ${ \phi ^ \alpha }$ satisfies the following saturation condition of the mixture,

\begin{align*}
{ \phi ^s} + { \phi ^w} + \mathop \sum \limits_{k = 1}^n { \phi ^k} = 1. \tag{6}
\end{align*}

The law of mass conservation requires that the velocities of the solid, water, and ionic phases must satisfy the continuity equation

\begin{align*}
{ \frac { \partial { \rho ^ \alpha } }  { \partial t } } + \nabla \cdot \left( { { \rho ^ \alpha } { { \bf { v } } ^ \alpha } } \right) = 0 \tag { 7 } 
\end{align*}

where ${{ \bf{v}}^ \alpha }$ is the velocity of component *α*.

The total density of the mixture, that is, the tissue, is

\begin{align*}
\rho = \mathop \sum \limits_{ \alpha = s , w , k} { \rho ^ \alpha } = \mathop \sum \limits_{ \alpha = s , w , k} { \phi ^ \alpha } \rho _T^ \alpha . \tag{8}
\end{align*}

From Equations (4) to (7), assuming the true densities are constant, the continuity equation for the mixture can be written as follows:

\begin{align*}
\nabla \cdot \left( {{ \phi ^s}{{ \bf{v}}^s} + { \phi ^w}{{ \bf{v}}^w} + {{ \bf{v}}^k} \mathop \sum \limits_{k = 1}^n { \phi ^k}} \right) = 0. \tag{9}
\end{align*}

The electroneutrality condition is as follows:

\begin{align*}
{z^F}{c^F} + \mathop \sum \limits_{k = 1}^n {z^k}{c^k} = 0 \tag{10}
\end{align*}

in which *z^k^* is the valence (including sign, positive for cations and negative for anions) of ion *k* and *c^F^* represents the fixed charge density (FCD). In Equation (10), the solid matrix is assumed to be negatively charged.

As in the triphasic theory, the fixed charges on the solid phase are conserved, so the FCD is a function of tissue deformation and water volume fraction (porosity). Thus,

\begin{align*}
{ \frac { \partial ( { \phi ^w } { c^F } ) }  { \partial t } } + \nabla \cdot \left( { { \phi ^w } { c^F } { { \bf { v } } ^s } } \right) = 0 \tag { 11 } 
\end{align*}

or it can be expressed with the tissue deformation as follows:

\begin{align*}
{ c^F } = { \frac { c_o^F }  { 1 + { \rm { tr } } \left( { \bf { E } } \right) / \phi _o^w } } \tag { 12 } 
\end{align*}

and the solidity of the tissue is expressed as

\begin{align*}
{ \phi ^s } = { \frac { \phi _o^s }  { 1 + { \rm { tr } } \left( { \bf { E } } \right) } } \tag { 13 } 
\end{align*}

where **E** is the strain tensor measured from the physicochemical reference configuration corresponding to a hypertonic salt bath. Since the values of ${ \phi ^k}$ are small in magnitude compared to ${ \phi ^s}$ and ${ \phi ^w}$, the saturation condition (6) can be written as ${ \phi ^s}$ + ${ \phi ^w}$* =* 1, and we have the following:

\begin{align*}
{ \phi ^w } = 1 - { \frac { \phi _o^s }  { 1 + { \rm { tr } } \left( { \bf { E } } \right) } } \tag { 14 } 
\end{align*}

where $c_o^F$, $\phi _o^s$, and $\phi _o^w$ represent the FCD, and the volume fractions of solid and water phases in the reference configuration, respectively.

If the gravity and magnetic effects are neglected, the gradients of the chemical and electrochemical potential, respectively, are the only driving forces, balanced by the frictional forces between different phases for their movement. The momentum equations for the tissue, water, anion, and cation are as follows:

\begin{align*}
\nabla \cdot \sigma = 0 \tag{15}
\end{align*}

\begin{align*}
- { \rho ^ \alpha } \nabla { \mu ^ \alpha } + {K^ \alpha } = 0 \tag{16}
\end{align*}

where ${K^ \alpha }$can be written as

\begin{align*}
{K^ \alpha } = \mathop \sum \limits_{ \beta = s , w , k}^n {f_{ \alpha \beta }} \left( {{{ \bf{v}}^ \beta } - {{ \bf{v}}^ \alpha }} \right) , \quad \quad \left( { \alpha = s , w , k} \right) \tag{17}
\end{align*}

Here, *σ* is the total stress of the mixture (tissue) and *μ^α^* is the electrochemical potential of phase *α*, respectively. The parameters ${f_{ \alpha \beta }}$ are the frictional coefficients per unit tissue volume between the interdiffusing *α* and *β* components. Following the classical mixture theories, it is assumed that ${f_{ \alpha \beta }} = {f_{ \beta \alpha}}$ holds in consistence with the Onsager reciprocity relations.^45,46^

If both the body and the inertial forces are neglected, the governing equations of the multiphasic model can then be derived as follows:

\begin{align*}
\sigma = - P{ \bf{I}} + { \lambda _s}{ \rm{tr}} \left( { \bf{E}} \right) { \bf{I}} + 2{ \mu _s}{ \bf{E}} \tag{18}
\end{align*}

\begin{align*}
{ \mu ^w } = \mu _o^w + \frac { 1 }  { { \rho _T^w } } \left( { P - RT \mathop \sum \limits_ { k = 1 } ^n { \varphi ^k } { c^k } + { B_w } { \rm { tr } } \left( { \bf { E } } \right) } \right) \tag { 19 } 
\end{align*}

\begin{align*}
{ \mu ^k } = \mu _o^k + { \frac { RT }  { { M^k } } } { \rm { ln } } \left( { { \gamma _k } { c^k } } \right) + { z^k } { \frac { { F_c } \psi }  { { M^k } } } . \tag { 20 } 
\end{align*}

Here, *R* is the universal gas constant, *F_c_* is the Faraday constant, ${ \gamma _k}$ are the activity coefficients of *k*th ionic species, $\mu _o^ \alpha \left( { \alpha = w , k} \right)$ are the chemical potential of phase *α* at the reference configuration, **I** is the identity tensor, ${ \varphi ^k}$ is the osmotic coefficient of ion *k*, and *B_w_* is the coupling coefficient.

The laws for linear electrokinetic transduction in isotropic media^47,48^ can be used to relate the relative fluid velocity **U** and the current density ${{ \bf{I}}_e}$ in the tissue to the gradients in fluid pressure *P* and electrical potential $\psi$ without considering the effects of diffusion potential,^42^

\begin{align*}
{ \bf{U}} = - {k_{11}} \nabla P + {k_{12}} \nabla \psi
\end{align*}

\begin{align*}
{{ \bf{I}}_e} = {k_{21}} \nabla P - {k_{22}} \nabla \psi \tag{21}
\end{align*}

where ${k_{11}}$ is the “short-circuit” hydraulic permeability, ${k_{12}}$ and ${k_{21}}$ are the electrokinetic coupling coefficients that are equal (${k_{12}} = {k_{21}}$) by the Onsager reciprocity theorem,^45,46^ and ${k_{22}}$ is the electrical conductivity. The coupling coefficients can be expressed in terms of the FCD or *ζ*-potential of the cartilage extracellular matrix using the microscopic continuum models for cartilage.^49^ The FCD is negative for materials like cartilage, so ${k_{12}}$ and ${k_{21}}$ are defined negative.

Inversion of Equation (21) leads to the expressions for gradients of pressure and potential,

\begin{align*}
\nabla P = - {b_{11}}{ \bf{U}} + {b_{12}}{{ \bf{I}}_e}
\end{align*}

\begin{align*}
\nabla \psi = {b_{21}}{ \bf{U}} - {b_{22}}{{ \bf{I}}_e} \tag{22}
\end{align*}

where ${b_{11}}$ is the (open circuit) Darcy hydraulic resistivity, ${b_{12}}$ and ${b_{21}}$ are the coupling coefficients that are equal (${b_{12}} = {b_{21}}$) according to the Onsager reciprocity theorem,^45,46^ and ${b_{22}}$ is the (no flow) electrical resistivity.

If the change in electric potential is required, then Equation (21) can be reformulated to obtain the following:

\begin{align*}
\nabla \psi = { k_e } \nabla P - \frac { 1 }  { { { k_ { 22 } } } } { { \bf { I } } _e } 
\end{align*}

\begin{align*}
{ \bf{U}} = - k \nabla P = {k_i}{{ \bf{I}}_e} \tag{23}
\end{align*}

where $k = {k_{11}} - ( {k_{12}}{k_{21}} / {k_{22}}$), ${k_e} = {k_{21}} / {k_{22}}$, and ${k_i} = {k_{12}} / {k_{22}}$. Note that *k* is the open-circuit hydraulic (Darcy) permeability.

For the case of triphasic materials, the relative water volume flux ${{ \bf{J}}^w}$ and ionic molar flux ${{ \bf{J}}^k}$ are defined as follows^50^:

\begin{align*}
{{ \bf{J}}^w} = { \phi ^w} \left( {{{ \bf{v}}^w} - {{ \bf{v}}^s}} \right) \tag{24}
\end{align*}

and

\begin{align*}
{{ \bf{J}}^k} = { \phi ^k}{c^k} \left( {{{ \bf{v}}^k} - {{ \bf{v}}^s}} \right) \tag{25}
\end{align*}

where the fluid velocity ${{ \bf{v}}^w}$ and ion velocity ${{ \bf{v}}^k}$ are defined as

\begin{align*}
{{ \bf{v}}^w} = - \left[ {{ \rho ^w} \nabla { \mu ^w} + { \rho ^ + } \nabla { \mu ^ + } + { \rho ^ - } \nabla { \mu ^ - }} \right] / {f_{ws}} \tag{26}
\end{align*}

\begin{align*}
{{ \bf{v}}^ + } = - \left[ {{ \rho ^w} \nabla { \mu ^w} + { \rho ^ + } \left( {1 + {f_{ws}} / {f_{w + }}} \right) \nabla { \mu ^ + } + { \rho ^ - } \nabla { \mu ^ - }} \right] / {f_{ws}} \tag{27}
\end{align*}

\begin{align*}
{{ \bf{v}}^ - } = - \left[ {{ \rho ^w} \nabla { \mu ^w} + { \rho ^ + } \nabla { \mu ^ + } + { \rho ^ - } \left( {1 + {f_{ws}} / {f_{w - }}} \right) \nabla { \mu ^ - }} \right] / {f_{ws}}. \tag{28}
\end{align*}

Similarly, the electrical current density inside the tissue assuming *k* ion species is

\begin{align*}
{{ \bf{I}}_e} = {F_c}{ \varphi ^w} \left[ { \mathop \sum \limits_{k = 1}^n {z^k}{c^k}{{ \bf{v}}^k} + {z^F}{c^F}{{ \bf{v}}^s}} \right] = {F_c} \mathop \sum \limits_{k = 1}^n {z^k}{{ \bf{J}}^k} \tag{29}
\end{align*}

or it can also be written as

\begin{align*}
{{ \bf{I}}_e} = {F_c} \left( {{{ \bf{J}}^ + } - {{ \bf{J}}^ - }} \right) = {F_c}{ \phi ^w} \left[ {{c^ + } \left( {{{ \bf{v}}^ + } - {{ \bf{v}}^s}} \right) - {c^ - } \left( {{{ \bf{v}}^ - } - {{ \bf{v}}^s}} \right) } \right] . \tag{30}
\end{align*}

Thus, from Equations (5), (10), (24), and (30), the relations for water flux and the electrical current density are,^50^

\begin{align*}
{{ \bf{J}}^w} = - {k_{11}} \nabla P + {k_{12}} \nabla \psi
\end{align*}

\begin{align*}
{{ \bf{I}}_e} = {k_{21}} \nabla P - {k_{22}} \nabla \psi \tag{31}
\end{align*}

where the values of the coefficients can be expressed as follows, while neglecting osmotic and strain effects:

\begin{align*}
{ k_ { 11 } } = { \frac { - { { \left( { { \phi ^w } } \right) } ^2 } }  { { f_ { ws } } } } 
\end{align*}

\begin{align*}
{ k_ { 12 } } = { k_ { 21 } } = - { c^F } { F_c } { \frac { { { \left( { { \phi ^w } } \right) } ^2 } }  { { f_ { ws } } } } 
\end{align*}

\begin{align*}
{ k_ { 22 } } = - { \left( { { F_c } { \phi ^w } } \right) ^2 } \left[ { { \frac { { { \left( { { c^ + } } \right) } ^2 } }  { { f_ { w + } } } } + { \frac { { { \left( { { c^F } } \right) } ^2 } }  { { f_ { ws } } } } + { \frac { { { \left( { { c^ - } } \right) } ^2 } }  { { f_ { w - } } } } } \right] . \tag { 32 } 
\end{align*}

If the quadphasic mixture theory is considered, it consists of four components: solid (*s*), water (*w*), cations (+), and anions (−). There are two phases: a solid (*s*) and a fluid (*f*) phase. A distinction is made here between the water and fluid. In this case, the fluid phase consists of three components: the water, the cations, and the anions. The relationships for the relative fluid and ion fluxes to the gradients in fluid pressure, ion concentrations, and the electrical potential are as follows^39,51^:

\begin{align*}
\begin{split}{{ \bf{J}}^w} = - { \bf{K}} \big [ { \nabla P + \big ( {{ \varphi ^w} - { \varphi ^ + }} \big ) RT \nabla {c^ + }} \\{ + \big ( {{ \varphi ^w} - { \varphi ^ - }} \big ) RT \nabla {c^ - } + {z^F}{c^F}{F_c} \nabla \psi } \big ]\end{split}
 \tag{33}
\end{align*}

\begin{align*}
{ { \bf { j } } ^k } = - { { \bf { D } } ^k } \left[ { \left( { 2 - { \varphi ^k } } \right) \nabla { c^k } + { z^k } { \frac { { F_c } }  { RT } } { c^k } \nabla \psi + { \frac { { M^k } }  { RT } } { c^k } \nabla P } \right] , \tag { 34 } 
\end{align*}

where ${{ \bf{J}}^w} = { \phi ^w} \left( {{{ \bf{v}}^w} - {{ \bf{v}}^s}} \right)$ and ${{ \bf{j}}^k} = {c^k} \left( {{{ \bf{v}}^k} - {{ \bf{v}}^f}} \right)$ are the relative fluid and ion fluxes, respectively, **K** is the hydraulic permeability tensor, ${{ \bf{D}}^k}$ is the diffusion tensor of ion *k*, and ${ \varphi ^w}$ is the osmotic coefficient of the water component.

### Triphasic theory with diffusion potential

The magnitude and polarity of the resultant electric potential for the cartilage tissue depend on the relative magnitudes of the streaming potential and diffusion potential. The streaming potential is caused by a pressure gradient (hydraulic and/or osmotic), while the diffusion potential arises by the gradients of mobile ions in the presence of an FCD gradient.^42^

As the FCD of cartilage tissue is nonuniform (intrinsic and/or induced by matrix deformation), so for the studies involving the mechano-electrochemical responses of the cartilage, the existence of a diffusion potential should not be neglected.

If this effect is also considered, Equation (31) for the linear electrokinetic transduction becomes as follows^40,42^:

\begin{align*}
{ { \bf { J } } ^w } = - { k_o } \nabla P - \mathop \sum \limits_ { k = 1 } ^n { b_k } \nabla \left( { { \frac { RT }  { { z^k } { F_c } } } { \rm { ln } } \left( { { \gamma _k } { c^k } } \right) } \right) - { \chi _o } \nabla \psi \tag { 35 } 
\end{align*}

\begin{align*}
{ { \bf { I } } _e } = - { g_o } \nabla P - \mathop \sum \limits_ { k = 1 } ^n { g_k } \nabla \left( { { \frac { RT }  { { z^k } { F_c } } } { \rm { ln } } \left( { { \gamma _k } { c^k } } \right) } \right) - { \chi _o } \nabla \psi \tag { 36 } 
\end{align*}

where *k_o_*, *b_k_*, *g_o_*, *g_k_*, and ${ \chi _o}$ are material parameters that are functions of the ion concentrations and the frictional coefficients ${f_{ \alpha \beta }}$ between *α* and *β* constituents.

When external circuits are not provided for the tissue to sustain a net charged flow of ions and electrons, the tissue is in a state of zero current. For such cases, at every point in the tissue, the sum of three currents must vanish, that is, ${{ \bf{I}}_e}$* =* Convection Current + Diffusion Current + Conduction Current = 0 or, from Equation (36), the gradient of the electric potential become as follows:

\begin{align*}
\nabla \psi = \left[ { - { g_o } \nabla P - \mathop \sum \limits_ { k = 1 } ^n { g_k } \nabla \left( { { \frac { RT }  { { z^k } { F_c } } } ln \left( { { \gamma _k } { c^k } } \right) } \right) } \right] / { \chi _o } . \tag { 37 } 
\end{align*}

For this case, there is no externally applied electric potential; the potential is entirely induced by the convection current and the diffusion current. This induced potential generates a conduction current to oppose the convection and diffusion currents to achieve the zero-current condition.

### Modified triphasic theory

An equivalent formulation for the triphasic theory has been proposed, which allows easy implementation for the finite element (FE) analysis.^52^ By using a simple transformation, the modified electrochemical/chemical potential functions are defined as follows:

\begin{align*}
{ \varepsilon ^w } = { \frac { \rho _ { \rm { T } } ^w \left( { { \mu ^w } - \mu _o^w } \right) }  { RT } } = \frac { P }  { { RT } } - \varphi \left( { { c^ + } - { c^ - } } \right) + { \frac { { B_w } }  { RT } } { \rm { tr } } \left( { \bf { E } } \right) \tag { 38 } 
\end{align*}

\begin{align*}
{ \varepsilon ^ + } = { \rm { exp } } \left[ { { \frac { { M^ + } \left( { { \mu ^ + } - \mu _o^ + } \right) }  { RT } } } \right] = { \gamma _ + } { c^ + } { \rm { exp } } \left( { { \frac { { F_c } \psi }  { RT } } } \right) \tag { 39 } 
\end{align*}

\begin{align*}
{ \varepsilon ^ - } = { \rm { exp } } \left[ { { \frac { { M^ - } \left( { { \mu ^ - } - \mu _o^ - } \right) }  { RT } } } \right] = { \gamma _ - } { c^ - } { \rm { exp } } \left( { - { \frac { { F_c } \psi }  { RT } } } \right) . \tag { 40 } 
\end{align*}

With the above definitions, the solid displacement ${{ \bf{u}}^s}$ (for infinitesimal deformation, it is related to the solid matrix velocity ${{ \bf{v}}^s}$ as ${{ \bf{v}}^s}$* = ∂*${{ \bf{u}}^s}$/*∂t*) and modified chemical potentials ${ \varepsilon ^w}$ and ${\varepsilon ^k}$ compose the four primary unknowns of the system; all the other unknowns can be derived from them. Multiplying Equations (39) and (40),

\begin{align*}
{ \varepsilon ^ + }{ \varepsilon ^ - } = { \gamma _ + }{ \gamma _ - }{c^ + }{c^ - }. \tag{41}
\end{align*}

Substituting Equation (41) in the electroneutrality Equation (10),

\begin{align*}
{ c^ + } = \frac { 1 }  { 2 } \left( { { c^F } + \sqrt { { { \left( { { c^F } } \right) } ^2 } + { \frac { 4 { \varepsilon ^ + } { \varepsilon ^ - } }  { { \gamma _ + } { \gamma _ - } } } } } \right) \tag { 42 } 
\end{align*}

\begin{align*}
{ c^ - } = \frac { 1 }  { 2 } \left( { - { c^F } + \sqrt { { { \left( { { c^F } } \right) } ^2 } + { \frac { 4 { \varepsilon ^ + } { \varepsilon ^ - } }  { { \gamma _ + } { \gamma _ - } } } } } \right) . \tag { 43 } 
\end{align*}

Similarly, from Equations (39) and (40), the electric potential is,

\begin{align*}
\psi = { \frac { RT }  { 2 { F_c } } } { \rm { ln } } \left( { { \frac { { \gamma _ - } { c^ - } { \varepsilon ^ + } }  { { \gamma _ + } { c^ + } { \varepsilon ^ - } } } } \right) . \tag { 44 } 
\end{align*}

With the triphasic momentum Equations (15) and (16), all the fluxes can be expressed with the combination of the gradients of the chemical potentials as follows:

\begin{align*}
{ { \bf { J } } ^w } = - \frac { { RT { \phi ^w } } }  { \xi } \left( { \nabla { \varepsilon ^w } + { \frac { { c^ + } }  { { \varepsilon ^ + } } } \nabla { \varepsilon ^ + } + { \frac { { c^ - } }  { { \varepsilon ^ - } } } \nabla { \varepsilon ^ - } } \right) \tag { 45 } 
\end{align*}

\begin{align*}
\begin{split}{{ \bf{J}}^ + } = - {{RT{ \phi ^w}{c^ + }} \over \xi
} \nabla { \varepsilon ^w} - {{RT{ \phi ^w}{c^ + }{c^ - }} \over {
\xi { \varepsilon ^ - }}} \nabla { \varepsilon ^ - } \\- \left[
{{{{ \phi ^w}{c^ + }{D^ + }} \over {{ \varepsilon ^ + }}} + {{RT{
\phi ^w}{{ \left( {{c^ + }} \right) }^2}} \over { \xi {
\varepsilon ^ + }}}} \right] \nabla { \varepsilon ^ + }\end{split}
 \tag{46}
\end{align*}

\begin{align*}
\begin{split}{{ \bf{J}}^ - } = - {{RT{ \phi ^w}{c^ - }} \over \xi } \nabla { \varepsilon ^w} - {{RT{ \phi ^w}{c^ + }{c^ - }} \over { \xi { \varepsilon ^ + }}} \nabla { \varepsilon ^ + } \\- \left[ {{{{ \phi ^w}{c^ - }{D^ - }} \over {{ \varepsilon ^ - }}} + {{RT{ \phi ^w}{{ \left( {{c^ - }} \right) }^2}} \over { \xi { \varepsilon ^ + }}}} \right] \nabla { \varepsilon ^ - }.\end{split}
 \tag{47}
\end{align*}

In the above derivation, the following relations have been used^48,53^:

\begin{align*}
{f_{sw}} = \; \xi { \phi ^w}
\end{align*}

\begin{align*}
{ f_ { + w } } = { \frac { RT { \phi ^w } { c^ + } }  { { D^ + } } } 
\end{align*}

\begin{align*}
{ f_ { - w } } = { \frac { RT { \phi ^w } { c^ - } }  { { D^ - } } } \tag { 48 } 
\end{align*}

where $\xi$ is the drag coefficient between the solid and water phase, and ${D^ + }$ and ${D^ - }$ are the diffusivities of the cation and anion, respectively.$\xi$, ${D^ + }$, and ${D^ - }$ were assumed to be constants. In the triphasic theory, the hydraulic permeability *k* is related to $\xi$ by

\begin{align*}
k = \frac { { { \phi ^w } } }  { \xi } = { \frac { { { \left( { { \phi ^w } } \right) } ^2 } }  { { f_ { ws } } } } \tag { 49 } 
\end{align*}

and ${f_{ + s}}$, ${f_{ - s}}$, ${f_{ + - }}$, and ${f_{ - + }}$ are neglected.^39,54^ With the four primary unknowns and the definitions of fluxes, the governing equations can be written as follows:

\begin{align*}
\nabla \cdot \sigma = 0 \tag{50}
\end{align*}

\begin{align*}
\nabla \cdot {{ \bf{v}}^s} + \nabla \cdot {{ \bf{J}}^w} = 0 \tag{51}
\end{align*}

\begin{align*}
{ \frac { \partial { \phi ^w } { c^ + } }  { \partial t } } + \nabla \cdot { { \bf { J } } ^ + } + \nabla \cdot \left( { { \phi ^w } { c^ + } { { \bf { v } } ^s } } \right) = 0 \tag { 52 } 
\end{align*}

\begin{align*}
{ \frac { \partial { \phi ^w } { c^ + } }  { \partial t } } + \nabla \cdot { { \bf { J } } ^ - } + \nabla \cdot \left( { { \phi ^w } { c^ - } { { \bf { v } } ^s } } \right) = 0. \tag { 53 } 
\end{align*}

All flux quantities must be calculated relative to the porous-permeable solid matrix. Using the triphasic momentum equations and writing the fluxes with combination of the gradients of chemical potentials, that is, using Equation (30),^38,52^ the electrical current density can be expressed with the modified electrochemical potentials as follows:

\begin{align*}
{ { \bf { I } } _e } = \left( { - { \frac { { \phi ^w } { c^ + } { D^ + } }  { { \varepsilon ^ + } } } \nabla { \varepsilon ^ + } + { \frac { { \phi ^w } { c^ - } { D^ - } }  { { \varepsilon ^ - } } } \nabla { \varepsilon ^ - } \; } \right) + { c^F } { { \bf { J } } ^w } . \tag { 54 } 
\end{align*}

By considering the triphasic constitutive equations, the electrical current density can be expressed as follows:

\begin{align*}
\begin{split}{{ \bf{I}}_e} = { \phi ^w} \left( {{D^ - } \nabla {c^ - }  - {D^ + } \nabla {c^ +  }} \right) \\- {{{F_c}} \over {RT}}{ \phi ^w} \left( {{c^ + }{D^ + }  - {c^ - }{D^ -  }} \right) \nabla \psi + {c^F}{{ \bf{J}}^w}.\end{split}
 \tag{55}
\end{align*}

In the above equation, the three terms are the diffusion current due to the ion concentration gradients, the conductive current due to the electrical potential gradient, and the streaming current due to the water convection, respectively. Under a condition with no current, ${{ \bf{I}}_e} = 0$, the electrical potential gradient can be expressed as

\begin{align*}
\nabla \psi = { \rm { \; } } { \frac { RT }  { { F_c } } } { \frac { { D^ - } \nabla { c^ - } - { D^ + } \nabla { c^ + } }  { { \phi ^w } \left( { { c^ + } { D^ + } + { c^ - } { D^ - } } \right) } } + { \frac { RT }  { { F_c } } } { \frac { { c^F } }  { { \phi ^w } \left( { { c^ + } { D^ + } + { c^ - } { D^ - } } \right) } } \tag { 56 } 
\end{align*}

that is, the electrical potential $\psi$ consists of two components, the diffusion potential due to the ion concentration gradients and the streaming potential due to the water convection. This is consistent with the existing literature on this subject,^48^ which is the most recent to our knowledge, although it dates back to 1965.

## Electrical Properties of Cartilage for *In Silico* Studies

**FIG. 2.**
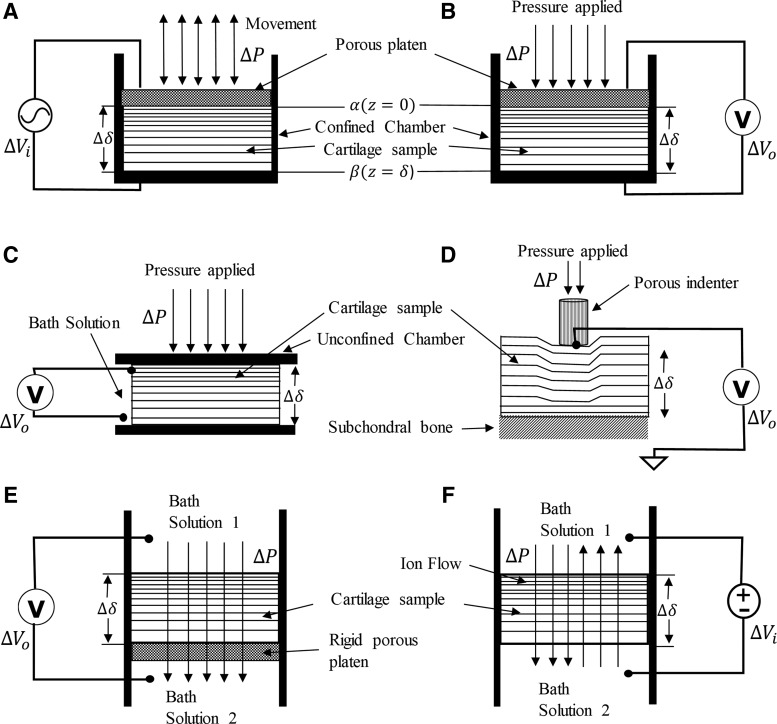
Schematic representation showing various approaches to observe the electrical properties in cartilage tissue. **(A)** Electrical stimulation, **(B)** confined compression, **(C)** unconfined compression, **(D)** indentation, **(E)** permeation configuration, **(F)** electro-osmosis/diffusion (without *V_i_*). Δ*P* is the change in pressure, Δ*δ* is the change in thickness of the cartilage sample, Δ*V_i_* and Δ*V_o_* are the change in input and output voltages, respectively.

The electromechanical properties of articular cartilage arise from the electrically charged nature of the tissue and its depth-dependent properties. There exists a unique environment in which chondrocytes are exposed to multiple biophysical cues.^55^ The extracellular matrix of the cartilage and its associated interstitial water, solutes, and ions are considered a signal transducer receiving input in the form of biophysical stimuli generating different signals for the growth and maintenance of the tissue.^56^ Various configurations used for *in silico* investigation of electrical properties of the cartilage have been schematically represented in Figure 2. In this section, we summarize the electrical properties of cartilage due to different biophysical stimuli as described in literature.

### Electrical stimulation

To our knowledge, only eight *in silico* studies are available in literature where the cartilage tissue has been discussed with regard to direct ES.^18,43,54,57–61^ Frank and Grodzinsky formulated a continuum model for the one-dimensional (1D) linear electrokinetic transduction in a bovine cartilage sample. They derived analytical expressions for the streaming potential and the current induced by oscillatory, uniaxially confined compression of the tissue. Furthermore, they deduced expressions for the mechanical stress generated by a current density or potential difference applied to the tissue and compared these results to their own experimental results.^42^ Later, Sachs and Grodzinsky extended this problem with two-dimensional applied current density instead of 1D and considered the cartilage tissue that was finite in thickness, while infinite in length.^60^ Furthermore, Kojic *et al.* reproduced the same experimental results numerically using an FE package for structural analysis (PAK).^58,59^ Ateshian *et al.*^61^ simulated the current-generated stress phenomenon in FEBio^62^ using the triphasic material theory,^38^ but they did not compare their results to the experimental study reported by Frank and Grodzinsky.^18^

Gu *et al.*^54^ studied the cartilage fluid flow and ion transport effects under an applied streaming current or potential using the triphasic theory. In another study, they reported the transport of interstitial water and ions in charged hydrated soft tissue like cartilage using a continuum mixture model, while also considering the electrical effects.^43^ Furthermore, they derived the well-known Hodgkin-Huxley equation for the resting potential of the cell membrane and investigated the phenomenon of electro-osmotic flow in charged hydrated soft tissue.^43^ Finally, Levenston *et al.*^57^ proposed a finite deformation theory for the analysis of confined compression configuration for a disk of cartilage and studied the electrokinetic phenomenon for the cartilage tissue.

### Mechanical stimulation

The application of mechanical stimulation generates electrical streaming potentials.^30^ There are *in silico* studies available where electrical properties of the cartilage tissue have been investigated due to mechanical stimulation. Lee *et al.*^63^ measured simultaneously the dynamic streaming potential amplitude with the stiffness for a disk of normal articular cartilage in oscillatory confined compression over a frequency range of 0.001–20 Hz relevant to impact loading. Then, theoretical and measured values of the streaming potential and stiffness were compared.^63^

Kim *et al.*^64^ analyzed the streaming potential behavior of cartilage in unconfined compression and they compared to their analytical poroelastic model, which can predict the oscillatory streaming potential, stiffness, and the spatial profiles of the physical stimuli. Similarly, Chen *et al.*^65^ theoretically predicted the streaming potential response of cartilage in the confined compression creep configuration and then tested these predictions through experiments in normal and proteoglycan-depleted tissue using biphasic theory along with the laws of linear electrokinetic transduction. In another study, Chen *et al.*^66^ tested the full-thickness bovine articular cartilage by oscillatory confined compression superimposed on a static offset up to 45%. With that data fit, they obtained the electrokinetic coefficient assuming homogeneity.^66^

The objective of the study performed by Lai *et al.*^42^ was to determine the nature of electric fields inside an articular cartilage, while accounting for the effects of both streaming potential and diffusion potential. In the said study, the transient 1D confined compression, stress-relaxation problem (in an open-circuit condition) using the triphasic theory was discussed to calculate the compressive strain, electric potential, and the FCD inside the cartilage.^42^ Similarly, Lai *et al.*^67^ also provided a complete description of the electric field in and around a cell inside a layer of tissue in a 1D confined compression stress relaxation experiment incorporating the triphasic theory.

Frijns *et al.*^51,68^ using a mixed FE method and quadphasic theory, simulated the mechanical and electrical behavior of a cartilage substitute caused by a change in the mechanical load or by a chemical load. They showed that the estimated parameter values were in the same range as reported by other studies in literature for a uniaxially confined swelling and compression experiment.^51,68^

Further studies performed triphasic analysis of the articular cartilage explicitly incorporating the FCD and ions, both diffusion and streaming potential, under unconfined compression.^69,70^ This analysis provided new information regarding the mechano-electrochemical signal transduction behavior of the tissue.^69,70^ Li and Herzog^71^ developed an FE formulation of streaming potentials in articular cartilage and incorporated it into a fibril-reinforced model, and subsequently used it to simulate interactions between an arthroscopic probe and articular cartilage in a knee joint.

Wan *et al.*^72^ briefly discussed some *in silico* electrical properties of the cartilage tissue in an unconfined compression experiment. Sachs and Grodzinsky^73^ have also discussed the linear electrical properties in the nondestructive assessment of cartilage degeneration for the electromechanical spectroscopy of cartilage using a surface probe with applied mechanical displacement.

### Other stimuli

In literature, there are also few *in silico* studies available where electrical properties of the cartilage tissue have been investigated due to other stimuli, including permeation, osmosis, and convection. These studies were performed by Gu *et al.*^50,53,74,75^ and by Quenneville and Buschmann.^76^

In their first study, Gu *et al.*^50^ derived the expressions for streaming current and potential within the cartilage tissue under 1D steady permeation condition, as a function of the intrinsic triphasic material coefficients. After this, they performed a study in which the relationships describing the dependence of the streaming potential on the negative FCD of the tissue were derived analytically using the triphasic theory and its dependency was proven when compared to the experimental data on streaming potential obtained from bovine femoral cartilage.^53^ Later, in another study, Gu *et al.*^74^ analyzed the negative osmotic flows through charged hydrated cartilage tissue caused by an applied osmotic pressure gradient using the triphasic mixture theory, and they discussed the quantitative results of ion fluxes and electric potential across the tissue during diffusion process.

In their fourth study, Gu *et al.*^75^ analyzed a 1D dynamic permeation of a monovalent electrolyte solution through a negatively charged hydrated cartilaginous tissue using the triphasic mechano-electrochemical theory developed by Lai *et al.*^38^ as the constitutive model for the tissue. The spatial distributions of stress parameters, ion concentrations, electrical potential, and ion fluxes within and across the tissue were analyzed.^75^

Finally, Quenneville and Buschmann^76^ developed a model of electrolyte transport across a charged membrane and examined the distribution of electric potential and mobile ion concentrations in response to forced convection.

All the current stimulation techniques for the *in silico* investigation of electrical properties of the cartilage described are summarized in the Tables 1–3 for the electrical, mechanical, and other stimulations, respectively, along with the respective mathematical equations, which have been noted in this article to describe these properties.

**Table 1.** Electrical Stimulation Studies

*Study*	*Theory*	*Diffusion potential*	*Kinematics*	*Configuration type*	*Equations*
Frank *et al.* 1987^18^	Biphasic	×	Electrokinetics	Confined compression	21
Sachs and Grodzinsky^60^	Biphasic	×	Electrokinetics	Unconfined compression	22
Kojic *et al.*^59^	Biphasic	×	Electrokinetics	Confined/unconfined compression	21
Kojic *et al.*^58^	Biphasic	×	Electrokinetics	Confined compression	21
Ateshian *et al.*^61^	Multiphasic	✓	Electrokinetics	Confined compression	35
Gu *et al.*^53^	Triphasic	×	Electrokinetics	Electro-osmosis	21
Gu *et al.*^43^	Multiphasic	✓	Continuity equation	Electro-osmosis	35 and 36
Levenston *et al.*^57^	Poroelastic	×	Electrokinetics	Confined/unconfined compression	21

**Table 2.** Mechanical Stimulation Studies

*Study*	*Theory*	*Diffusion potential*	*Kinematics*	*Configuration type*	*Equations*
Lee *et al.*^63^	Biphasic	×	Electrokinetics	Confined compression	21
Kim *et al.*^64^	Biphasic	×	Electrokinetics	Unconfined compression	23
Chen *et al.*^65^	Biphasic	×	Electrokinetics	Confined compression	21
Chen *et al.*^66^	Biphasic	×	Electrokinetics	Confined compression	21
Lai *et al.*^42^	Triphasic	✓	Electrokinetics	Permeation/confined compression	35 and 36
Lai *et al.*^67^	Triphasic	✓	Continuity equation	Confined compression	36
Frijns *et al.*^51^	Quadphasic	✓	Electrokinetics	Confined compression	33 and 34
Frijns *et al.*^68^	Quadphasic	✓	Electrokinetics	Confined compression	33 and 34
Mow *et al.*^17^	Triphasic (modified)	✓	Continuity equation	Unconfined compression	55
Sun *et al.*^70^	Triphasic (modified)	✓	Continuity equation	Unconfined compression	56
Li *et al.*^71^	Biphasic	×	Electrokinetics	Indentation	21
Sachs and Grodzinsky^73^	Biphasic	×	Electrokinetics	Spectroscopy	21
Wan *et al.*^72^	Triphasic	✓	Electrokinetics	Unconfined compression	21

**Table 3.** Other Stimulation Studies

*Study*	*Theory*	*Diffusion potential*	*Kinematics*	*Configuration type*	*Equations*
Gu *et al.*^50^	Triphasic	×	Electrokinetics	Permeation	31
Gu *et al.*^53^	Triphasic	×	Continuity equation	Permeation	31
Gu *et al.*^74^	Triphasic	✓	Electrokinetics	Negative osmosis	21
Gu *et al.*^75^	Triphasic (modified)	✓	Continuity equation	Permeation	56
Quenneville and Buschmann^76^	Nernst-Planck/Poisson eqs.	✓	Continuity equation	Convection	21

## One-Dimensional Electrokinetic Transduction Model Based on Continuum Theory

The electrokinetic model consists of electrical-to-mechanical and mechanical-to-electrical transductions as shown in Figure 2A and B, respectively. We analyzed the 1D electrokinetic transduction in a charged, homogeneous, isotropic, hydrated bovine cartilage sample and compared the analytical and FE solutions using FEniCS with the experimental data from the studies reported by Frank and Grodzinsky.^18,34^ We extracted data points using the software WebPlotDigitizer.^77^

We combined the laws for linear electrokinetic transduction^47^ in ionized media with linear biphasic theory for cartilage.^20^ A constitutive law for the total stress ${T_{ij}}$ in a homogeneous, isotropic tissue valid for small strains ${ \varepsilon _{ij}}$ is

\begin{align*}
{T_{ij}} = 2G \left( c \right) { \varepsilon _{ij}} + \left( { \lambda \left( c \right) { \varepsilon _{kk}} - P} \right) { \delta _{ij}} , \tag{57}
\end{align*}

where the Lamé constants *G* and *λ* are functions of the ionic content (*c*) of the cartilage, *P* is the fluid pressure, and the subscripts *i*, *j*, and *k* represent the axes.

We used the laws for linear electrokinetic transduction in isotropic media^47^ to relate the relative fluid velocity **U** and the current density ${{ \bf{I}}_e}$ in the tissue to the gradients in fluid pressure *P* and electrical potential $\psi$ already described by Equation (21).

For the case of incompressible fluid and solid constituents, continuity relates the fluid and solid velocities ${{ \bf{v}}^w}$ and ${{ \bf{v}}^s}$,^78^

\begin{align*}
{ \phi ^w} \nabla \cdot {{ \bf{v}}^w} + \left( {1 - { \phi ^w}} \right) \nabla \cdot {{ \bf{v}}^s} = 0 \tag{58}
\end{align*}

Since we are concerned with a confined uniaxial configuration, only deformations in the *z* direction have been considered described in Figure 2. After some mathematical steps, the equation of motion in the complex frequency domain reads as follows:

\begin{align*}
j \omega u = { H_A } k { \frac { \partial { \mu ^2 } }  { \partial { z^2 } } } + { k_i } { I_e } + { U_o } , \tag { 59 } 
\end{align*}

where ${H_A} = 2G + \lambda$ is the equilibrium confined compression modulus,^79^ *U_o_* is the constant of integration and ${I_e}$ is magnitude of the current density.

A general solution to the complex equation of motion (59) is given by

\begin{align*}
u \left( z \right) = { \frac { \left( { { u^ \alpha } - { u_J } } \right) { \rm { sinh } } \gamma \left( { \delta - z } \right) + \left( { { u^ \beta } - { u_J } } \right) { \rm { sinh } } \gamma z }  { { \rm { sinh } } \gamma \delta } } , \tag { 60 } 
\end{align*}

where

\begin{align*}
{u^ \alpha } = u \left( {z = 0} \right) \ and \ {u^ \beta } = u \left( {z = \delta } \right) , \tag{61}
\end{align*}

are the displacement amplitudes imposed at the top (*α*) and bottom (*β*) surfaces of the tissue as schematically illustrated in Figure 2, ${ \gamma ^2} = j \omega / {H_A}k$, ${u_J} = \left( {{k_i}J + {U_o}} \right) / j \omega$ and *ω* is the angular frequency.

Following the approach of Frank and Grodzinsky,^18^ first, we considered the sinusoidal streaming potential and dynamic stiffness of a cartilage sample in response to sinusoidal displacement under open-circuit condition (${{ \bf{I}}_e} = 0$). Then we considered the stress generated by a sinusoidal current density applied to the electrodes with the jaw-to-jaw displacement held fixed (*u =* 0).

Equation (60) is solved analytically to obtain the expression for dynamic stiffness (*Λ*), streaming potential (*V_o_*), and current-generated stress (*σ*)

\begin{align*}
\Lambda = { \frac { { \Lambda _s } { \Lambda _ { oc } } }  { { \Lambda _s } + { \Lambda _ { oc } } } } \tag { 62 { \rm a } } 
\end{align*}

\begin{align*}
{ V_o } = - { k_e } \left[ { { \frac { { \Lambda _s } { \Lambda _v } }  { { \Lambda _s } + { \Lambda _ { oc } } } } } \right] \frac { u }  { \delta } \tag { 62 { \rm b } } 
\end{align*}

\begin{align*}
\sigma = { \frac { - { k_i } { I_e } }  { j \omega \delta } } \left[ { { \frac { { \Lambda _s } { \Lambda _v } }  { { \Lambda _s } + { \Lambda _ { oc } } } } } \right] , \tag { 62 { \rm c } } 
\end{align*}

where ${ \Lambda _s}$ is the stiffness of porous platen, ${ \Lambda _{oc}} = {H_A} \delta { \rm{coth}} \gamma \delta$, and ${ \Lambda _v} = {H_A} \delta { \rm{tanh}} \left( { \gamma \delta / 2} \right)$.

**FIG. 3.**
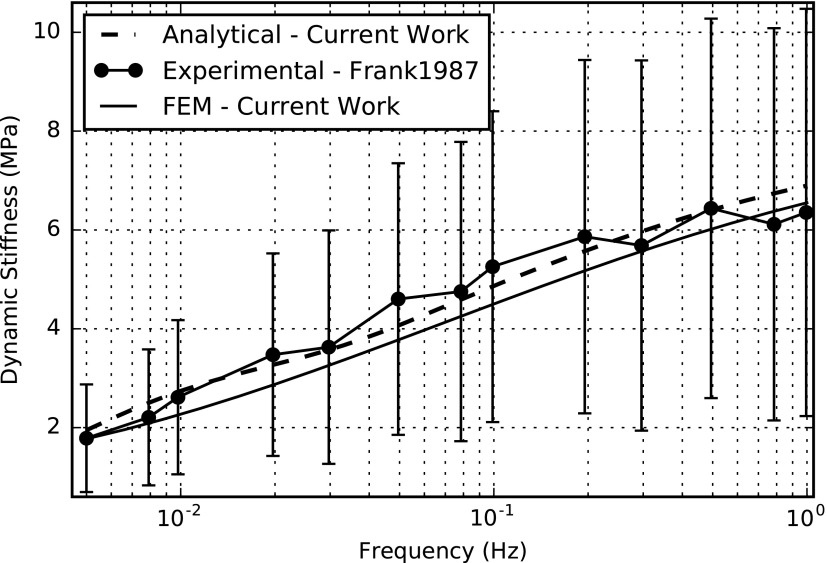
Mechanical-to-electrical transduction—amplitude of the dynamic stiffness versus frequency.

**FIG. 4.**
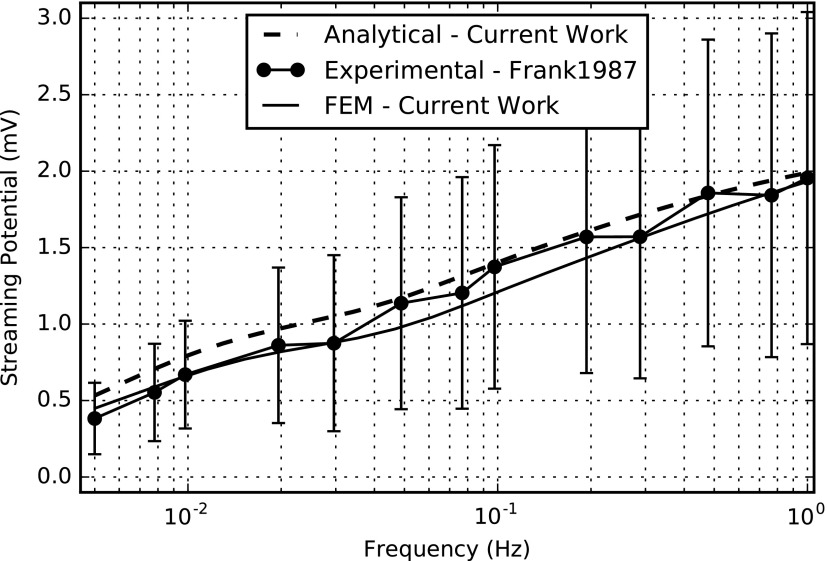
Mechanical-to-electrical transduction—amplitude of the streaming potential versus frequency.

**FIG. 5.**
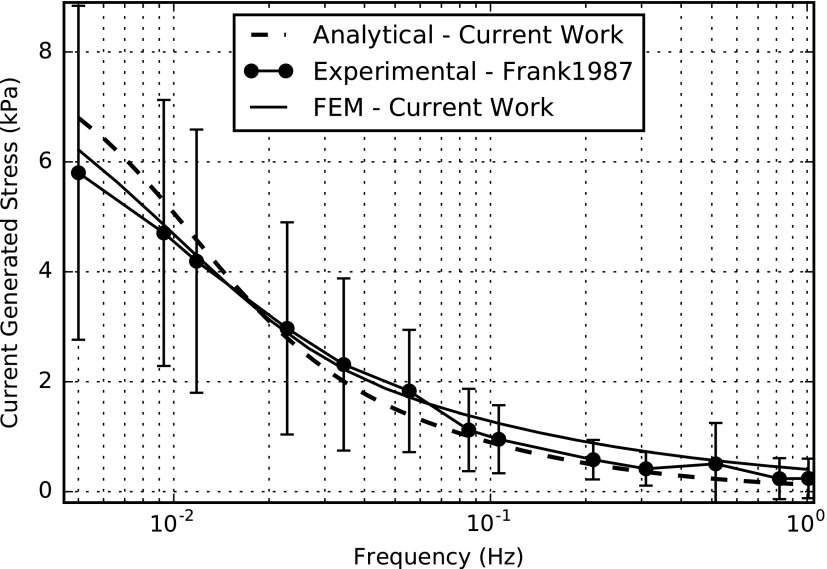
Electrical-to-mechanical transduction—amplitude of the current-generated stress versus frequency.

We performed the numerical solution of the partial differential equation (59) using the open source tool FEniCS together with a Python interface. The computation time was 0.65 s with 20,002 degrees of freedom (DOFs) on a computer with 32GB RAM, Intel(R) Xeon(R) CPU E5-1630 v3 @ 3.00 GHz. The FE simulation results for the amplitudes of dynamic stiffness, streaming potential, and current-generated stress for the frequency range of 0.005–1 Hz (relevant to impact loading) are shown in Figures 3–5, respectively. In the same figures, we compared the FE simulations to the analytical results and experimental data extracted from the experimental results of Frank and Grodzinsky.^34^

Table 4 lists all the parameters used for the simulations. The fit of theoretical values to the analytical and experimental values does not employ any adjustable parameter in contrast to the studies reported by Kojic *et al.*^18,58^ The frequency-dependent viscoelastic behavior of cartilage tissue is evident from these graphs.^36^ This open-source numerical implementation allows relatively easy implementation without any complex coding techniques.

**Table 4.** Principal Set of Parameters

Compressive modulus, *H_A_*	1 MPa
Platen stiffness, *Λ_s_*	8.3 MPa
Applied displacement, *u*	10 × 10^−6^ m
Thickness of sample, *δ*	680 × 10^−6^ m
Diameter of sample, *D*	6.35 × 10^−3^ m
Applied current density, *I_e_*	3.80 A/m^2^
Hydraulic permeability, *k*	3 × 10^−15^ m^2^/Pa·s
Electrokinetic constant, *k_i_*	−2.07 × 10^−8^ V/Pa
Electrokinetic constant, *k_e_*	−2.18 × 10^−8^ V/Pa

Our numerical simulation results compare well to the analytical and experimental results.^18,34^ Some small variations could be due to the fact that the actual experimental setup is in 3D, but the analysis only considers the amplitudes in 1D. In addition, the analysis only regarded *z*-directed amplitudes, while neglecting those in *x* and *y* directions, while in reality, there can be small contributions to the amplitudes from the other two directions as well.

**FIG. 6.**
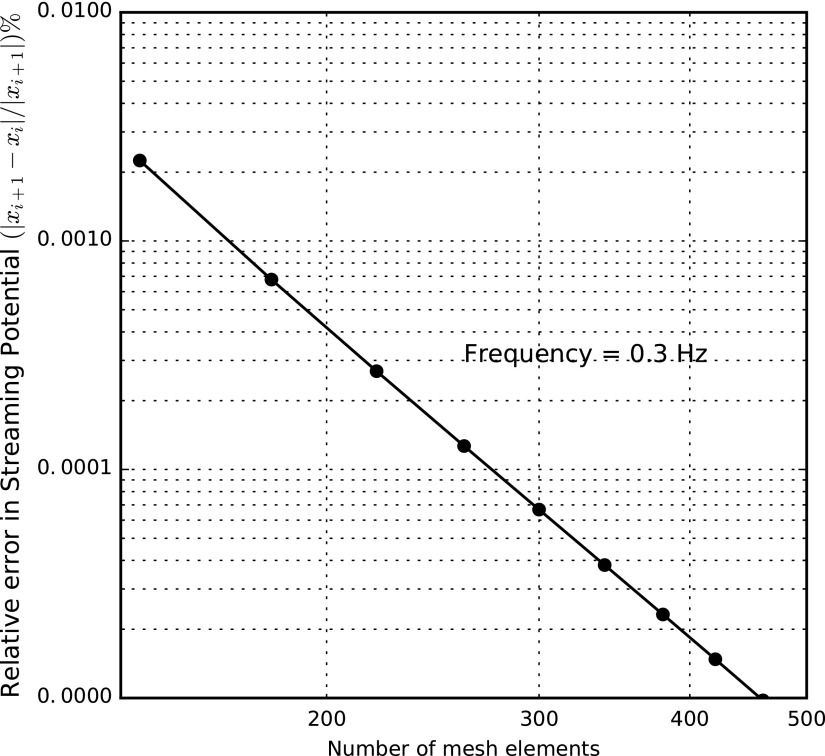
Relative error in amplitude of streaming potential as a function of mesh elements taken at a representative value of *f* = 0.3 Hz.

To determine the convergence of the FE analysis for mechanical-to-electrical transduction, we examined the relative error in the streaming potential at a representative frequency of 0.3 Hz, while refining the mesh. Figure 6 depicts the results of this convergence study. Obviously, the relative error in the amplitude of the streaming potential approaches zero if the mesh incorporates more than 460 elements, corresponding to more than 922 DoFs.

**FIG. 7.**
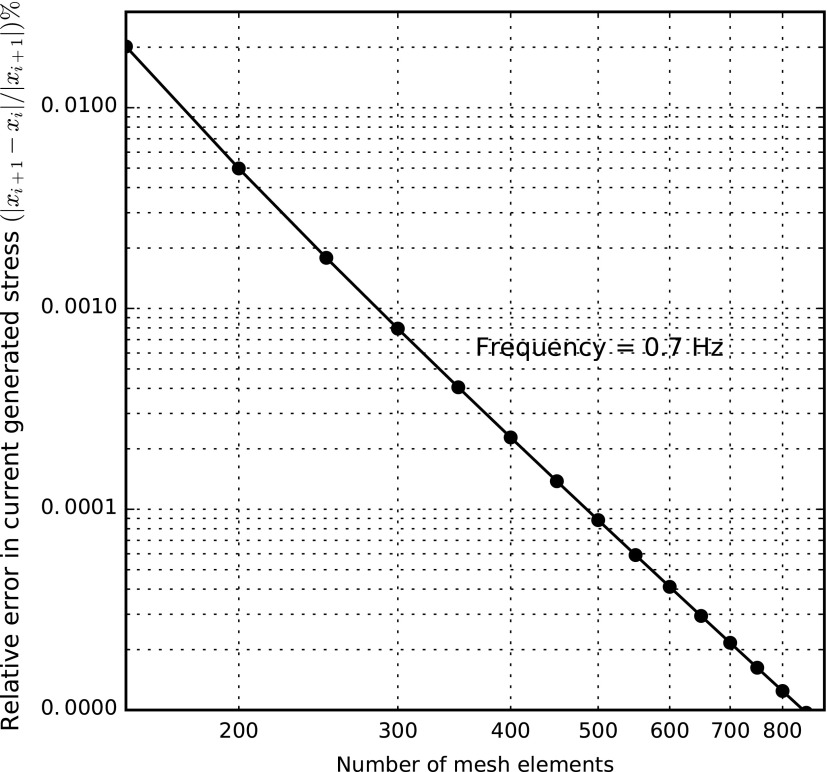
Relative error in amplitude of current-generated stress as a function of mesh elements taken at a representative value of *f* = 0.7 Hz.

Similarly, we analyzed the convergence of the FE analysis for the electrical-to-mechanical transduction for the current-generated stress at a representative frequency of 0.7 Hz. Figure 7 makes evident that the relative error in the amplitude of the current-generated stress approaches zero if the mesh incorporates more than 850 elements, corresponding to more than 1702 DoFs. Thereby, good mesh convergence is achieved with almost 460 and 850 elements for the mechanical-to-electrical and electrical-to-mechanical transductions, respectively.

## Conclusion

This work summarizes *in silico* investigations discussing the effects of different biophysical stimuli, that is, electrical, mechanical, and chemical stimuli, on the induced electrical properties and physiology of cartilage tissue. Clearly, biophysical stimuli in the form of electrical field resembling the intrinsic signals are applicable as an additional tool toward the development of optimal therapies for cartilage regeneration. Nevertheless, few models have been proposed to study the *in silico* induced electrical properties of cartilage at the tissue level due to direct ES, but a comprehensive and consistent model to observe the electrical interactions at the cellular level is still lacking. On the other hand, investigating the electrical properties of cartilage *in silico* due to indirect ES, that is, capacitive and inductive ES, is still an open research area. In this context, the presented open source implementation of the electrokinetic phenomena in a cartilage tissue sample using FEniCS can be useful to study the *in silico* electrical interactions at microscale as well as for modeling of the indirect ES for the cartilage tissue.
